# Conditional disruption of the *osterix* gene in chondrocytes during early postnatal growth impairs secondary ossification in the mouse tibial epiphysis

**DOI:** 10.1038/s41413-019-0064-9

**Published:** 2019-08-05

**Authors:** Weirong Xing, Catrina Godwin, Sheila Pourteymoor, Subburaman Mohan

**Affiliations:** 1 Musculoskeletal Disease Center, VA Loma Linda Healthcare System, Loma Linda, CA 92357 USA; 2 Department of Medicine, Loma Linda University, Loma Linda, CA 92357 USA; 3 Department of Orthopedics, Loma Linda University, Loma Linda, CA 92357 USA; 4 Department of Biochemistry, Loma Linda University, Loma Linda, CA 92357 USA

**Keywords:** Bone, Physiology

## Abstract

In our previous studies, we have found that the prepubertal increase in thyroid hormone levels induces osterix (Osx) signaling in hypertrophic chondrocytes to transdifferentiate them into osteoblasts. To test if *Osx* expressed in chondrocytes directly contributes to transdifferentiation and secondary ossification, we generated *Osx*^*flox/flox*^; *Col2-Cre-ERT2* mice and knocked out *Osx* with a single injection of tamoxifen at postnatal day (P) 3 prior to evaluation of the epiphyseal bone phenotype by µCT, histology, and immunohistochemistry (IHC) at P21. Vehicle (oil)-treated *Osx*^*flox/flox*^; *Col2-Cre-ERT2* and tamoxifen-treated, *Cre*-negative *Osx*^*flox/flox*^ mice were used as controls. µCT analysis of tibial epiphyses revealed that trabecular bone mass was reduced by 23% in the *Osx* conditional knockout (cKO) compared with control mice. Trabecular number and thickness were reduced by 28% and 8%, respectively, while trabecular separation was increased by 24% in the cKO mice. Trichrome staining of longitudinal sections of tibial epiphyses showed that bone area and bone area adjusted for total area were decreased by 22% and 18%, respectively. IHC studies revealed the presence of abundant *Osx*-expressing prehypertrophic chondrocytes in the epiphyses of control mice at P10, but not in the cKO mice. Furthermore, expression levels of MMP13, COL10, ALP, and BSP were considerably reduced in the epiphyses of cKO mice. We also found that *Osx* overexpression in ATDC5 chondrocytes increased expression of *Col10*, *Mmp13*, *Alp*, and *Bsp*. Our data indicate that Osx expressed in chondrocytes plays a significant role in secondary ossification by regulating expression of genes involved in chondrocyte hypertrophy and osteoblast transdifferentiation.

## Introduction

Bone formation is known to occur via two routes: intramembranous and endochondral ossification. In the intramembranous bone formation route, bone develops directly from sheets of mesenchymal connective tissue, which is formed by mesenchymal cells from the cranial neural crest, sclerotomes, and lateral plate mesoderm that migrate and proliferate. In the endochondral bone formation route, condensation of mesenchymal progenitor cells and their subsequent differentiation into chondrocytes lead to the establishment of cartilage, which is subsequently replaced by trabecular bone.^1,2^ There are two centers of ossification for endochondral ossification—a primary and a secondary center. The primary ossification center (POC) usually appears in the diaphysis of the long bones or in the body of the irregular bones during embryonic development, while the secondary ossification center (SOC) occurs in the epiphysis of long bones at the time of birth in mammals.^3^ Endochondral ossification at the POC is tightly regulated by a number of growth factors (PTHrP, Ihh, IGF-I, BMP/TGFβ, Wnt, and vascular endothelial growth factor (VEGF)) and transcription factors (Sox9, Runx2, Osterix (Osx), and β-catenin).^4–11^ Dysregulation in the production and/or actions of any of the factors that regulate endochondral ossification can result in skeletal diseases, including chondrodysplasias and osteoarthritis.^6,12^ While the processes leading to POC formation have been well established, signaling pathways that stimulate SOC formation are not well understood.

In our previous studies on the mechanisms for the thyroid hormone effect on bone formation, we focused on SOCs since the time of appearance of SOCs in some bones in several species, including mice, rats, and humans coincides with the time when peak levels of thyroid hormone are attained.^13–15^ In humans, the identification of proximal humeral epiphyseal ossification centers occurred around a gestational age of 38 weeks, which coincided with the attainment of peak levels of thyroid hormone (36–40 weeks). In rodents, the peak levels of thyroid hormone are attained at week 2 when SOC formation of the distal femur and proximal tibia also takes place. By using mouse models that are deficient in thyroid hormone and/or growth hormone, we found that endochondral ossification of the proximal tibia SOC is severely compromised due to thyroid hormone deficiency, and that thyroid hormone replacement for 10 days completely rescued this phenotype.^16^ However, SOCs in different bones are known to appear at different times in both humans and mice. These temporal association studies suggest that thyroid hormone is permissive for normal SOC formation and that lack of thyroid hormone results in delayed SOC formation in certain long bones.^17^ We also examined the expression levels of key regulators responsible for chondrocyte/osteoblast differentiation in the epiphyses by real-time PCR and found that *Osx* expression is severely compromised in the epiphyses of thyroid hormone-deficient mice at P10 when serum levels of thyroid hormone peak in mice.^16^ This reduced *Osx* mRNA level in hypothyroid *Tshr*^−/−^ mice is completely rescued by treatment of thyroid hormone-deficient mice with replacement doses of T3/T4 for 5 days. We further showed that thyroid hormone effects on *Osx* expression were mediated via activation of thyroid hormone receptor β1 signaling.^18,19^

OSX was initially identified as an osteoblast-specific transcription factor, and mice with total knockout of *Osx* function failed to form bone and died immediately after birth.^20^ In recent studies, however, we and others have shown that *Osx* is also expressed in chondrocytes and contributes to skeletal development.^18,19,21,22^ Based on the relative importance of OSX in osteoblast development and bone formation and our findings that OSX expression is severely compromised in the chondrocytes of thyroid hormone-deficient mice during secondary ossification, we proposed the hypothesis that OSX expressed in chondrocytes contributes to the thyroid hormone effects on chondrocyte differentiation and osteoblast development during postnatal growth. To test this hypothesis, we generated *Osx*-floxed mice with or without the *Col2a1-Cre-ERT2* transgene. We treated these mice with a single injection of tamoxifen at P3 to induce inactivation of the *Osx* gene in epiphyseal chondrocytes prior to evaluation of chondrocyte and osteoblast formation and trabecular bone formation in the epiphysis at 3 weeks of age.

## Results

### Conditional knockout of *Osx* expression in chondrocytes reduces the trabecular bone volume in the epiphysis

**Fig. 1:**
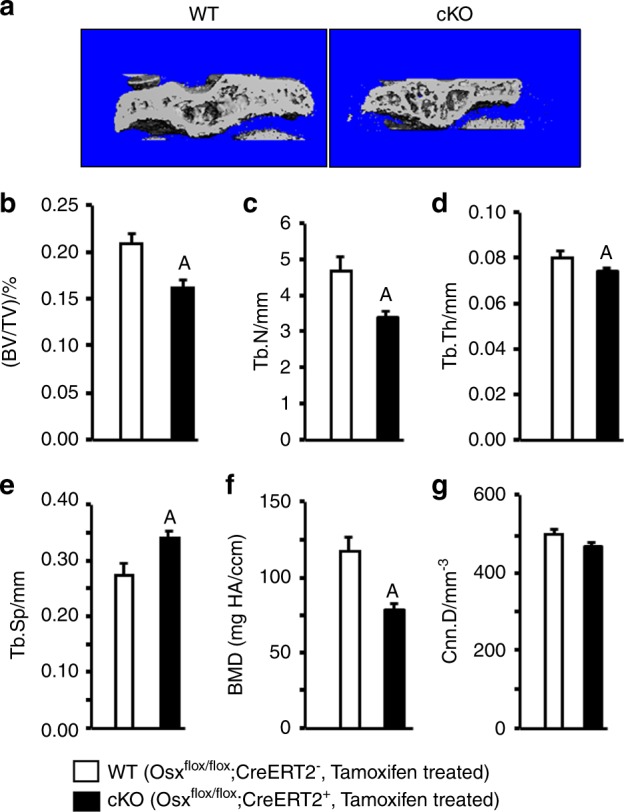
Conditional inactivation of osterix in epiphyseal chondrocytes reduces trabecular bone mass in the epiphyses in mice. **a** Representative µCT images of the tibial epiphyses of WT mice (*Os*x^flox/flox^; *Cre-ERT2*^−^ treated with tamoxifen) and *Osx* cKO mice (*Osx*^flox/flox^; *Cre-ERT2*^+^ treated with tamoxifen) (*N* = 7, 4 males and 3 females). **b**–**g** Quantitative µCT data of the trabecular bone volume to total volume (Tb. BV/TV), trabecular number (Tb. N), trabecular thickness (Tb. Th), trabecular separation (Tb. Sp), bone mineral density, and connectivity density of the tibial epiphyses as shown in **a**. Values are the mean ± SEM (*N* = 5, 3 males and 2 females). (A) Significant difference (*P* < 0.05) in *Osx* cKO epiphyses as compared with WT controls

**Fig. 2:**
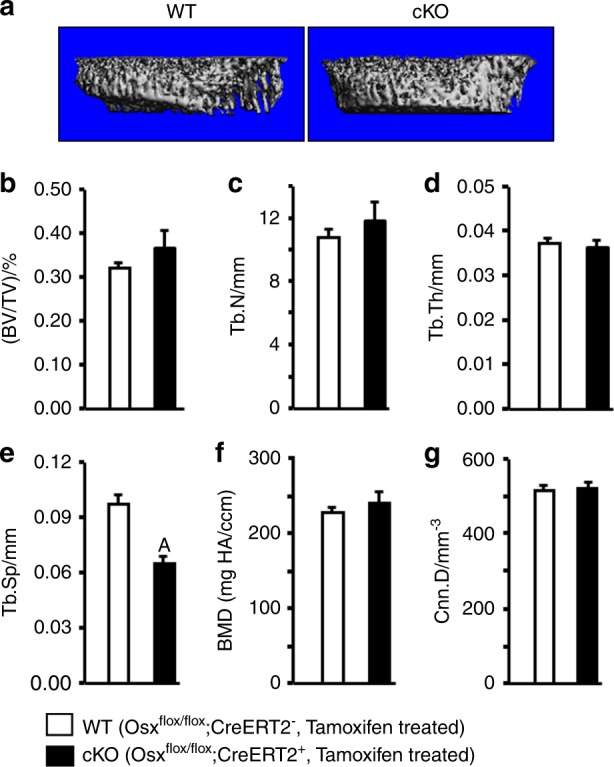
Conditional inactivation of osterix in epiphyseal chondrocytes reduces trabecular separation in the growth plate in mice. **a** Representative µCT images of the tibial growth plate of WT mice (*Osx*^flox/flox^; *Cre-ERT2*^+^ treated with corn oil) and *Osx* cKO mice (*Osx*^flox/flox^; *Cre-ERT2*^+^ treated with tamoxifen). **b**–**g** Quantitative µCT data of the trabecular bone volume to total volume (Tb. BV/TV), trabecular number (Tb. N), trabecular thickness (Tb. Th), trabecular separation (Tb. Sp), bone mineral density, and connectivity density of the tibial epiphyses as shown in **a**. Values are the mean ± SEM (*N* = 7, 4 males and 3 females). (A) Significant difference (*P* < 0.05) in *Osx* cKO epiphyses as compared with WT controls

To examine whether *Osx* expressed in chondrocytes mediates trabecular bone formation in the epiphysis, we generated mice with postnatal inactivation of the *Osx* gene in epiphyseal chondrocytes by breeding *Osx*-floxed mice with transgenic *Col2α1-Cre-ERT2* mice. *Cre*-negative, *Osx*-floxed control mice (WT) and *Cre*-positive, *Osx*-floxed homozygous mice (cKO) were given a single injection of tamoxifen at P3 to induce conditional knockout (cKO) of the *Osx* gene in chondrocytes. At P21, mice were euthanized, and their bones were used for µCT, histology, and IHC. As shown in Fig. 1a, µCT shows reduced trabecular bone in the tibial epiphysis of *Osx* cKO mice compared with WT control mice. Quantitative analysis revealed that bone volume/tissue volume (BV/TV) was significantly reduced by 24%, which was caused by a significant decrease in trabecular number (Tb. N) and thickness and an increase in trabecular separation (Tb. Sp) in the *Osx* cKO mice (Fig. 1b–e). Total vBMD was significantly reduced by 32% (Fig. 1f). The small reduction in connectivity density (Cnn. D) was not significant (Fig. 1g). Unlike bone phenotypes in the epiphysis, BV/TV, Tb. N, and trabecular thickness (Tb. Th) were unchanged in the secondary spongiosa of the tibias (Fig. 2a–d), but the Tb. Sp was significantly decreased by 27% (Fig. 2e). There were no changes in bone mineral density (BMD) and Cnn. D in the secondary spongiosa either (Fig. 2f, g).

To verify that the observed trabecular bone changes in the epiphyses of cKO mice are due to disruption of the Osx gene and not due to the effects of genotypes *per se*, we compared the epiphyseal bone phenotypes of *Cre*-positive, *Osx*-floxed mice treated with tamoxifen with *Cre*-positive, *Osx*-floxed mice treated with corn oil, and found a similar reduction in trabecular bone mass in the epiphyses of *Cre*-positive, *Osx*-floxed mice compared with the *Cre*-negative, *Osx*-floxed control mice treated with the same dose of tamoxifen (Fig. 1a–g, Supplementary data). Similarly, trabecular bone parameters were unaltered in the secondary spongiosa of the tibias, except that the Tb. Sp was significantly decreased by 27% (Fig. 2a–g, Supplementary data).

**Fig. 3:**
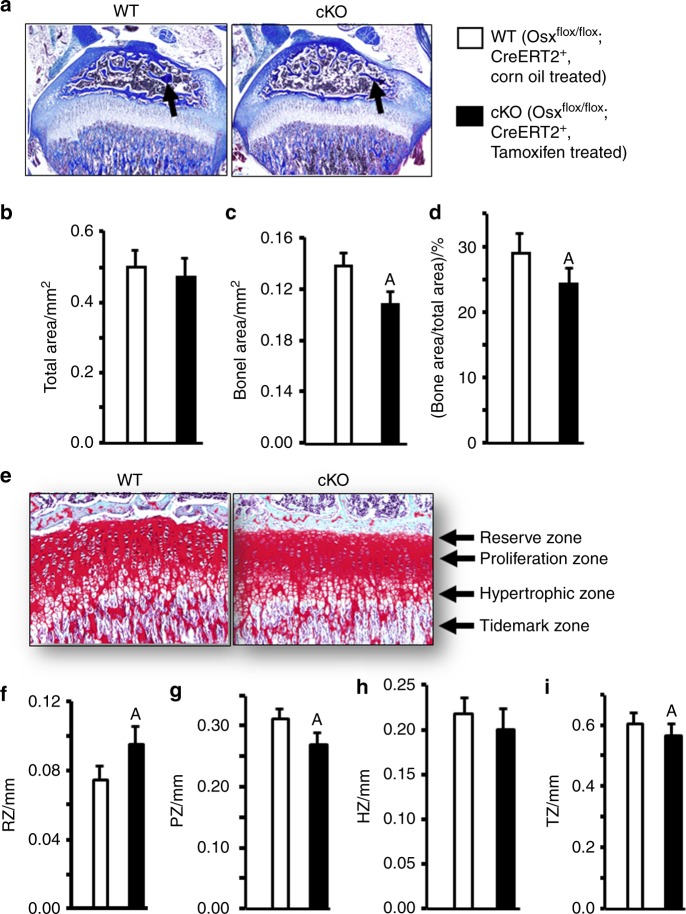
Conditional inactivation of osterix in epiphyseal chondrocytes delays bone formation in the secondary ossification center of the epiphyses in WT and cKO mice. **a** Trichrome staining images of tibial epiphysis sections of WT mice (*Osx*^flox/flox^; *Cre-ERT2*^+^ treated with corn oil) and *Osx* cKO mice (*Osx*^flox/flox^; *Cre-ERT2*^+^ treated with tamoxifen). **b**–**d** Quantitative histomorphometric data of the total area, bone area, and bone area/total area in A. **e** Safranin-O staining images of tibial growth plate sections of WT mice and *Osx* cKO mice. **f**–**i** Quantitative histomorphometric data of the reserve zone (RZ), proliferation zone (PZ), hypertrophic zone (HZ), and tidemark zone (TZ) of the growth plate in B. Values are the mean ± SEM (*N* = 6, 3 males and 3 females). (A) Significant difference (*P* < 0.05) in *Osx* cKO epiphyses as compared with WT controls

To confirm the reduced trabecular bone mass in the epiphyses of *Osx* cKO mice measured by µCT, we performed bone area measurements in trichrome-stained longitudinal sections of the epiphyses, which also revealed that conditional inactivation of *Osx* expression in chondrocytes impaired the SOC formation in the epiphyses of cKO mice (Fig. 3a). Bone area and adjusted bone area by total area in the epiphyses were significantly reduced by 21% and 20%, respectively, in the *Osx* cKO mice (Fig. 3b–d). Longitudinal sections of the tibial growth plates stained with Safranin-O revealed that the thickness of the reserve zone was increased by 30% in *Osx* cKO mice as compared with the WT mice (Fig. 3e, f). However, the thicknesses of the proliferation zone, hypertrophic zone, and tidemark zone were significantly reduced by 17%, 17%, and 8%, respectively, in the *Osx* cKO mice (Fig. 3g–i).

### Deficiency of OSX delays chondrocyte hypertrophy and conversion to osteoblasts in *Osx* cKO mice

**Fig. 4:**
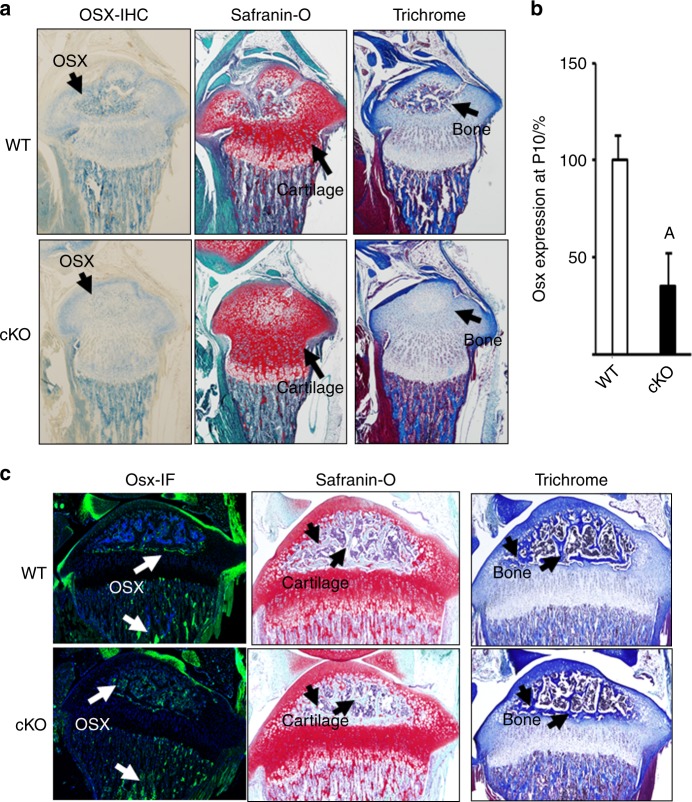
Conditional inactivation of osterix in epiphyseal chondrocytes reduces osterix expression and bone formation in the epiphyses in mice. **a** Longitudinal proximal tibial sections of WT mice (*Osx*^flox/flox^; *Cre-ERT2*^+^ treated with corn oil) and *Osx* cKO mice (*Osx*^flox/flox^; *Cre-ERT2*^+^ treated with tamoxifen) at P10 were immuno-stained with anti-OSX antibody (blue staining). Consecutive sections were also stained with Safranin-O and trichrome. **b** Reduced osterix expression in the epiphyses of cKO mice. RNA was extracted from epiphyses of WT and *Osx* cKO mice at postnatal day 10 and used for real-time PCR (*N* = 4 per group).^40^ **A** Significant difference (*P* < 0.05) in cKO versus WT mice. **c** The longitudinal sections of tibial epiphysis at P21 in the *Osx* cKO and WT control mice were stained with immunofluorescent anti-OSX antibody (green). Consecutive sections were also stained with Safranin-O and trichrome

To confirm that a single injection of tamoxifen was sufficient to induce *Cre*-mediated recombination of *Osx*-floxed alleles and reduce OSX expression in epiphyseal chondrocytes, we performed IHC in the longitudinal sections of tibial epiphysis at P10 in the *Osx* cKO and WT control mice. Figure 4a shows robust OSX expression in the mid epiphyses of WT mice, where active bone formation is taking place. However, OSX expression was severely compromised in the epiphyseal chondrocytes of *Osx* cKO mice, thus suggesting that a single injection of tamoxifen was sufficient to induce *Cre-ERT2*-mediated DNA recombination and reduce OSX expression. By contrast, OSX expression was not significantly affected in the osteoblasts of the primary or secondary spongiosa in *Osx* cKO mice (Fig. 4a).

Previously, we have shown that cartilage-to-bone conversion occurs during the second week of postnatal life in mice and that rising thyroid hormone levels are indispensable for this conversion. To determine if disruption of *Osx* expression in epiphyseal chondrocytes delays cartilage-to-bone conversion, we stained longitudinal sections of tibial epiphyses of *Osx* cKO and WT mice at P10 with Safranin-O and trichrome stains. Figure 4a shows that while cartilage-to-bone conversion occurred promptly in the epiphyses of WT mice as expected, it was compromised or delayed in the epiphyses of *Osx* cKO mice as evidenced by more Safranin-O-stained cartilage and less trichrome-stained ossified bone in *Osx* cKO epiphysis as compared with the WT control at this age. Expression of *Osx* in the epiphysis was reduced by 65% in the cKO mice at P10 as compared with the age-matched WT control mice (Fig. 4b). Consistent with the reduced expression of *Osx* after cKO, there was a considerable delay in the formation of hypertrophic chondrocytes and osteoblasts in *Osx* cKO mice at P10 that normalizes at 3 weeks of age (Fig. 4c).

**Fig. 5:**
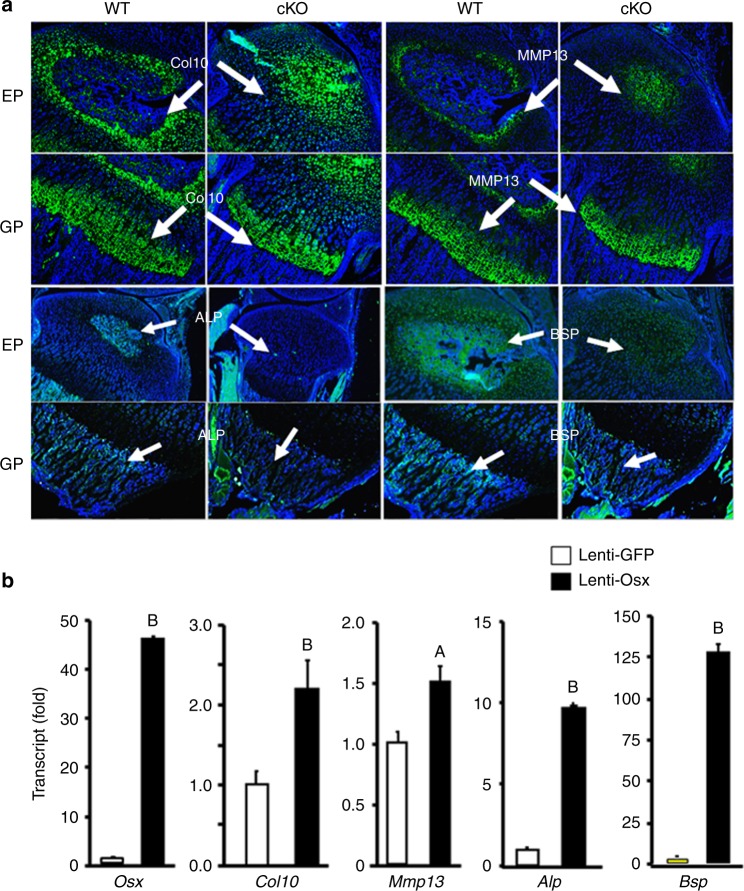
Conditional inactivation of osterix in epiphyseal chondrocytes delays secondary ossification center formation by reducing chondrocyte hypertrophy and conversion to osteoblasts. **a** Expression of hypertrophic chondrocyte markers collagen 10 (Col10) and MMP13 and osteoblast markers ALP and BSP in the tibial epiphyses of WT and cKO mice at 10 days of age. Longitudinal sections of the tibia were stained with immunofluorescent anti-Col10, anti-MMP13, anti-ALP, and anti-BSP antibodies. Expression of Col10, MMP13, and BSP in the tibial epiphyses (EP) and growth plates (GP) of WT and cKO mice were stained in green. **b** Overexpression of *Osx* induces Col10, MMP13, ALP, and BSP expression in ATDC5 chondrocytes. ATDC5 cells were transduced with lenti-*Osx* or lenti-GFP viral particles for 3 days, and total RNA was extracted for real-time RT-PCR. Values are the mean ± SEM (*N* = 4). (A) Significant difference in *Osx*-overexpressing cells (*P* < 0.05) compared with the corresponding GFP-expressing cells. (B) Significant difference in *Osx*-overexpressing cells (*P* < 0.01) compared with the corresponding GFP-expressing cells

To determine if the reduced endochondral ossification in the *Osx* cKO mice is due to reduced chondrocyte hypertrophy and conversion to osteoblasts, we measured expression levels of chondrocyte hypertrophic markers Col10 and MMP13 and osteoblast markers ALP and BSP in the tibial epiphyses of cKO and control 10-day-old mice. In the WT mice, MMP13 and Col10 expression was seen in the hypertrophic chondrocytes that were surrounding the newly formed bone (i.e., the periphery of the epiphysis), while ALP and BSP expression was detected in the osteoblasts of the newly formed bone (Fig. 5). By contrast, the expression of both MMP13 and Col10 markers in the *Osx* cKO mice was primarily seen in the middle of the epiphysis, and ALP and BSP expression in the epiphysis was severely compromised at P10. The signals for Col10 and MMP13 are strong in the growth plate hypertrophic chondrocytes, while BSP expression is strong in the osteoblasts of the epiphysis and primary spongiosa. Expression of ALP and BSP was not significantly altered in the osteoblasts of the primary or secondary spongiosa in *Osx* cKO mice as compared with the WT mice.

To investigate if reduction in OSX expression is the cause for changes in the expression of markers of hypertrophic chondrocytes and osteoblasts, we overexpressed OSX using a lentiviral vector and measured expression levels of COL10, MMP13, BSP, and ALP by real-time PCR. Figure 5b shows that *Osx* expression was increased by 45-fold in *Osx*-overexpressing ATDC5 chondrocytes compared with *GFP* overexpression control. While there was a modest increase in expression of Col10 and MMP13, expression of ALP and BSP were increased by 10- and 125-fold, respectively, in *Osx*-overexpressing cells compared with control cells that overexpressed GFP.

## Discussion

Previous studies have shown that the rising thyroid hormone level during the second week of postnatal life in mice is essential for initiation and progression of the SOC at the epiphysis.^18,19^ The cartilage in the epiphyses of the tibias was gradually converted into bone between P7 and P14 in the WT control mice when serum levels of thyroid hormone rise, but was delayed in the thyroid hormone-deficient mice. We have demonstrated that robust Osx expression occurred in the epiphyseal chondrocytes of WT, but not thyroid hormone-deficient mice during the period when cartilage-to-bone conversion occurs in the SOC.^18,19^ In this study, we used a transgenic approach to conditionally disrupt OSX expression in epiphyseal chondrocytes prior to initiation of the SOC to test the role of OSX in chondrocyte hypertrophy and osteoblast formation. Consistent with an established role of chondrocyte-produced OSX in primary ossification,^21,31^ our findings show that *Osx* expressed in chondrocytes plays a key role postnatally in chondrocyte hypertrophy and transdifferentiation into osteoblasts and, thereby, in cartilage-to-bone conversion during secondary ossification. However, we cannot exclude the possibility of involvement of Osx-positive-chondrocyte-derived factors in regulating osteoblast differentiation in an autocrine or paracrine manner. In this regard, a recent study demonstrated that Osx-positive hypertrophic chondrocyte-derived VEGF might regulate angiogenesis, osteoblast differentiation, and bone formation during embryonic development.^32,33^

OSX was initially identified as an osteoblast-specific transcription factor that activates a repertoire of genes during differentiation of preosteoblasts into mature osteoblasts and osteocytes.^20^ In *Osx* null mutant mice, no endochondral or intramembranous bone formation occurred due to the arrest in osteoblast differentiation.^20^ Since OSX was also known to be expressed, albeit at lower levels, in prehypertrophic and hypertrophic chondrocytes,^21,22,34^ we and others examined the role of OSX expressed in chondrocytes by conditional disruption of the *Osx* gene in Col2α1-expressing chondrocytes. Surprisingly, cKO of *Osx* in chondrocytes using Col2α1-Cre resulted in postnatal lethality because of respiratory insufficiency; the cKO embryos exhibited defective chondrocyte differentiation and bone formation. Heterozygous cKO mice also had skeletal defects.^31^ Body length and areal BMD of the total body, femur, and tibia were significantly reduced in mice with conditional disruption of one allele of *Osx* in chondrocytes. Histological analyses revealed that the impairment of longitudinal growth was associated with disrupted growth plates in the *Osx*^flox/+^; *Col2α1-Cre* mice. Primary chondrocytes isolated from cKO embryos showed reduced expression of hypertrophic chondrocyte markers.^31^ Based on these findings, we predicted a key role for OSX expressed in epiphyseal chondrocytes in new bone formation that occurs in the epiphysis. To address this prediction, we generated mice with postnatal inactivation of the *Osx* gene in epiphyseal chondrocytes by breeding *Osx*-floxed mice with transgenic *Cre-ERT2* mice in which tamoxifen-activatable Cre expression is under the control of the *Col2α1* promoter. Our data show that postnatal inactivation of *Osx* in chondrocytes via a single injection of tamoxifen at P3 resulted in reduced endochondral ossification. The trabecular bone mass of tibial epiphyses analyzed by µCT was reduced by 24% in the *Osx* cKO compared with control mice, which was caused by significant reductions in Tb. N and thickness and an increase in Tb. Sp. Our histological and IHC analyses reveal that the reduced trabecular bone in the tibial epiphyses of *Osx* cKO mice is due to delayed cartilage-to-bone conversion caused by a delay in the development of hypertrophic chondrocytes and their conversion to osteoblasts. Consistent with a role for OSX in transdifferentiation of chondrocytes into osteoblasts, we found that overexpression of OSX caused a robust increase in the expression of osteoblast-specific markers (Fig. 5b). In addition, we found that knockdown of OSX in ATDC5 chondrocytes impaired expression of osteoblast markers.^34^ These and our recently published data on the lineage mapping of Col2α1-expressing chondrocytes during epiphyseal bone formation provide direct evidence for a role for OSX expressed in chondrocytes in endochondral ossification of epiphyses via a mechanism involving chondrocyte-to-osteoblast transdifferentiation.

In terms of the question of why the trabecular bone phenotype of mice with postnatal inactivation of the *Osx* gene in chondrocytes is much less severe in this study compared with our earlier study involving KO of the *Osx* gene in chondrocytes throughout embryonic development, there are several potential explanations. In this study, we opted to knock out the *Osx* gene in *Osx*-floxed mice that express *Cre-ERT2* in chondrocytes via a single injection of tamoxifen at P3 based on our earlier data that thyroid hormone levels start to rise at P5 followed by an increase in *Osx* expression in the epiphysis.^18,19^ It is possible that a single dose of tamoxifen was not sufficient to disrupt the *Osx* gene in all Col2α1-expressing epiphyseal chondrocytes while the mice with cartilage-specific Osx ablation lack Osx throughout embryonic development. In addition, a study published by Newton et al.^35^ reveals evidence that a stem-cell niche develops postnatally in the epiphyseal growth plate and provides a continuous supply of chondrocytes. Thus, osteogenic cells derived from mesenchymal stem cells after P3 are likely to express OSX because of the specificity of the Cre driver used. In our future studies, we will monitor the *Cre-*mediated excision of loxP sites at multiple time points after tamoxifen administration and inject tamoxifen at multiple times during the period when secondary ossification occurs to disrupt the *Osx* gene in chondrocytes throughout this period.

Tamoxifen can act as a weak estrogen receptor agonist and produce an estrogen-like effect on bone.^36^ The kinetics of ER fusion protein activation in vivo have been investigated in recent studies.^37^ These studies have shown that the half-life of tamoxifen after a single intraperitoneal administration is about 12 h–16 h and that the efficacy of tamoxifen to induce *Cre-*mediated recombination of target genes is more effective after multiple tamoxifen administrations than administration of a single dose. In a recent study, tamoxifen treatment in mice at a dose of 100 mg/kg/day for 4 consecutive days significantly increased trabecular BV 1 month after the last tamoxifen injection.^38^ In our study, trabecular BV was reduced in the *Osx* cKO mice. Furthermore, we compared epiphyseal bone phenotypes of tamoxifen-treated, *Osx*-floxed mice (cKO) with those of WT mice and found a similar reduction in trabecular BV, thus suggesting the decreased BV in the *Osx* cKO mice is due to *Osx* gene disruption and not due to a tamoxifen effect. Furthermore, the trabecular bone phenotype measured by µCT was not different between tamoxifen-treated WT mice and oil-treated, *Cre*-negative, *Osx*-floxed mice, thus suggesting that a single dose of tamoxifen at 200 µg/mouse (40 mg/kg) was not effective in producing estrogen-like effects on trabecular bone in the epiphysis.

Chondrocyte differentiation is regulated by a coordinated balance of positive and negative signals from various transcription factors including activators, repressors, coactivators, and corepressors on the chromatin template. One master positive regulator of endochondral ossification is *Osx*.^18,19,31^ In terms of the mechanism by which *Osx* controls endochondral ossification, a previous study has demonstrated that MMP13 is a direct target of OSX, as the MMP13 promoter contains OSX response elements.^21^ Overexpression of MMP13 in OSX-deficient limb bud cells stimulated the calcification of chondrocyte matrices, and presence of an MMP13 inhibitor blocked Osx-induced calcification of the matrices in the growth plate.^21^ In chondrocyte-specific MMP13 KO mice, chondrogenic matrices accumulated in the hypertrophic zone of the growth plate.^39^ Consistent with these observations, our data showed that MMP13 and Col10 matrix proteins were markedly lower in the epiphyses of *Osx* cKO mice than those of control WT mice. The reduction in the expression of MMP13 and Col10 is associated with impaired SOC formation, strongly indicating that degradation of cartilaginous collagen by OSX-induced MMP13 is also required for the bone formation of the SOC in the epiphysis.

While our findings demonstrate an important role for OSX in chondrocyte hypertrophy in the long bone epiphysis during SOC formation, the causal role of OSX in mediating thyroid hormone effects on epiphyseal bone formation remains to be established. Our future work will cross hypothyroid (*hyt/hyt*) mice with chondrocyte-specific *Osx* cKO mice to generate mice that are homozygous for *hyt* and *Osx*-floxed alleles and are either Col2-CreER positive or negative to test if conditional disruption of the *Osx* gene in chondrocytes blocks the thyroid hormone effect on SOC formation in hypothyroid mice. If the prediction that chondrocytes are an important source of osteoblasts in bone formation processes during bone growth and remodeling, and that increased OSX in these chondrocytes is an important regulatory step in chondrocyte-to-osteoblast transdifferentiation, turns out to be true, then further understanding of the mechanisms of this cell transformation could provide exciting new strategic approaches to develop anabolic therapies for osteoporosis and other bone-wasting diseases.

## Materials and methods

### Chemicals, cell lines, and biological reagents

The chondrogenic cell line ATDC5 derived from teratocarcinoma AT805 was purchased from the American Type Culture Collection (Manassas, VA). Antibodies used for immunohistochemistry (IHC) are listed in detail in Table 1 (Supplementary data).

### Generation of conditional Osx-knockout mice

*Col2a1-Cre-ERT2* transgenic mice were purchased from the Jackson Laboratory.^23^ *Osx*-floxed mice in which exon 2 of the *Osx* gene was flanked by *loxP* sites were generated in Dr Benoit de Crombrugghe’s laboratory at the University of Texas MD Anderson Cancer Center in Houston.^24^ Mice with postnatal inactivation of the *Osx* gene in epiphyseal chondrocytes were generated by breeding *Osx*-floxed homozygous, *Cre-*negative mice with *Osx*-floxed homozygous, *Cre-ERT2*-positive mice in which *Cre* expression is under the control of the *Col2α1* promoter. Our breeding schedule generated 50% *Cre-*negative *loxP* homozygous mice and 50% *Cre-*positive *loxP* homozygous mice all of which were used in the experiments. *Cre*-positive or *Cre*-negative *Osx*-floxed homozygous mice were given a single injection of 0.2 mg of tamoxifen (40 mg·kg^−1^) at postnatal day 3 (P3) to activate Cre recombinase and induce conditional inactivation of the *Osx* gene in chondrocytes. At P21, mice were euthanized, and bones used for µCT, histology, and IHC. Oil-treated, *Cre*-positive, *Osx*-floxed mice and tamoxifen-treated, *Cre*-negative, *Osx*-floxed mice were used as controls. DNA extracted from tail snips was used for PCR-based genotyping. Mice were housed at the VA Loma Linda Healthcare System Veterinary Medical Unit (Loma Linda, CA) under standard approved laboratory conditions. All the procedures were performed with the approval of the Institutional Animal Care and Use Committees of the VA Loma Linda Healthcare System. Mice were anesthetized with approved anesthetics (isoflurane, ketamine/xylazine) prior to procedures. For euthanasia, animals were exposed to CO_2_ prior to cervical dislocation.

### µCT evaluation of the SOCs and the growth plates

Trabecular bone microarchitecture of the tibial epiphyses (i.e., the SOC) and proximal metaphyses isolated from 21-day-old mice was assessed by µCT (viva CT40, Scanco Medical AG, Switzerland) as described previously.^25,26^ The tibias were fixed in 10% formalin overnight, washed with PBS, and immersed in PBS to prevent them from drying. The bone was scanned by X-ray at 55 kVp at a resolution of 10.5 µm/slice. The scout view of the whole leg, including the tibial epiphysis and proximal metaphysis, was used for analyses. To analyze SOC formation, the proximal tibial epiphyses were used for measurement of newly formed bone. Parameters such as BV (mm^3^), bone volume fraction (BV/TV, %), Tb. N (mm^−1^), Tb. Th (mm), Tb. Sp (mm), trabecular BMD (HA/ccm), and Cnn. D (mm^−3^) were evaluated as described previously.^25–27^

### IHC and immunofluorescence analyses

IHC was performed using a rabbit IHC kit (Vector Laboratories, Burlingame, CA) according to the manufacturer’s instruction. Briefly, tibial epiphyseal sections were deparaffinized in HistoChoice clearing agent (Sigma-Aldrich), rehydrated in a graded series of ethanol and tap water, and treated with 3% H_2_O_2_ for 30 min to inactivate endogenous peroxidase activity. The sections were then rinsed thoroughly with PBS (pH 7.4) and digested with hyaluronidase in PBS (10 mg·ml^−1^) at 37 °C for 30 min for epitope recovery. The sections were pretreated with a blocking solution containing normal goat serum for 20 min and then incubated with primary antibody specific to OSX at a dilution of 1:200 as shown in Table 1 (Supplementary data). Negative control sections were incubated with normal rabbit or mouse IgG. After an overnight incubation at 4 °C, the sections were rinsed with PBS and incubated with biotinylated anti-rabbit secondary antibodies for 30 min at room temperature. The sections were then washed in PBS, incubated with VECTASTAIN Elite ABC Reagent for 30 min, rinsed again with PBS, and incubated with the Vector Blue substrate until the desired color stain developed. Similarly, immunofluorescence was carried out using Vector kits DI-1788 for green and DI-1794 for red for polyclonal antibodies generated from rabbit and a vector MOM kit BMK-2202 for monoclonal antibodies generated in mice (Vector Laboratories, Burlingame, CA) according to the manufacturer’s instructions. Nuclei were counterstained with DAPI (100 ng·ml^−1^) for 10 min.

### Viral plasmid construction, lentivirus generation, and transduction

The lentiviral pRRLsin-cPPT-SSFV-*Osx*-wpre plasmid was generated by replacing GFP with a PCR product corresponding to the mouse *Osx sequence* using the *Sgf1* and *Pmel* restriction sites of the pRRLsin-cPPT-SSFV-GFP-wpre vector. Lentiviral particles were generated by co-transfection of pRRLsin-cPPT-SSFV-*Osx*-wpre plasmid or pRRLsin-cPPT-SSFV-GFP-wpre control plasmid with Pax2 and VSVG plasmids in 293 T cells as described previously.^28,29^ Forty-eight hours after transfection with FuGene, culture supernatants containing viral particles were collected, spun at 2 000 × *g* for 10 min, and filtered through a 0.45-mm filter. Titers were determined by infecting 293 T cells with serial dilutions and examining GFP expression of infected cells 24 h after infection. ATDC5 cells were transduced by adding Lenti-GFP or Lenti-Osx viral supernatant at a multiplicity of infection of 5 in the presence of polybrene (8 mg·mL^−1^) for 24 h followed by replacement of fresh DMEM/F12 medium containing 10% FBS, penicillin (100 units/mL), and streptomycin (100 μg·mL^−1^). Forty-eight hours later, cultures were harvested for RNA extraction and real-time PCR analyses.

### RNA extraction and quantitative PCR

RNA was extracted from ATDC5 cells or bones as described previously.^25^ The epiphyses and growth plate regions of long bones were isolated and ground to powder in liquid nitrogen using a mortar and pestle prior to RNA extraction.^30^ An aliquot of RNA (25 ng) was reverse-transcribed with an oligo(dT)_12−18_ primer into cDNA in a 20 µL reaction volume. The real-time PCR reaction contained 0.5 µL of template cDNA, 1× SYBR GREEN master mix (ABI), and 100 nmol·L^−1^ of specific forward and reverse primers in a 12 μL reaction volume. Primers for peptidyl prolyl isomerase A were used to normalize the expression data for the genes of interest. The primer sequences used for real-time PCR are listed in Table 2 (Supplementary data).

### Statistical analysis

Data are presented as mean ± standard error of the mean (SEM) from 6 to 10 mice for each group. Significant differences were determined as *P* < 0.05 or *P* < 0.01. Data were analyzed by Student’s *t*-test or two-way ANOVA as appropriate. Because we used prepubertal mice (21 days or younger) for all our experiments and because gender differences are manifested only after puberty (around 5–6 weeks of age), we pooled data from both genders in all our analyses.

## Supplementary information

supplementary table 1

supplementary table 2

supplementary figure 3

supplementary figure 4

supplementary figure 5
