# Supplementary material for: Conditional disruption of the osterix gene in chondrocytes during early postnatal growth impairs secondary ossification in the mouse tibial epiphysis
Source: Bone Res. 2019 Aug 5;7:24. doi: 10.1038/s41413-019-0064-9 (PMC6804621; doi:10.1038/s41413-019-0064-9)
Supplement: Supplementary file 1 — supplementary table 1 [file 41413_2019_64_MOESM1_ESM.docx]

**Supplementary table 1. Primer sequences used for real time PCR**

| **Gene** | **Forward primer** | **Reverse primer** |
| --- | --- | --- |
| *Ppia* | 5’-CCATGGCAAATGCTGGACCA-3’ | 5’-TCCTGGACCCAAAACGCTCC-3’ |
| *Alp* | 5’ ATGGTAACGGGCCTGGCTACA | 5’-: AGTTCTGCTCATGGACGCCGT |
| *Bsp* | 5’- AACGGGTTTCAGCAGACAACC | 5’- TAAGCTCGGTAAGTGTCGCCA |
| *Col10* | 5’- ACGGCACGCCTACGATGT | 5’- CCATGATTGCACTCCCTGAA |
| *Mmp-13* | 5’- CATCCATCCCGTGACCTTAT | 5’- TCATAACCATTCAGAGCCCA |
| *Osx* | 5’- AGAGGTTCACTCGCTCTGACGA | 5’- TTGCTCAAGTGGTCGCTTCTG |
