# Supplementary material for: Conditional disruption of the osterix gene in chondrocytes during early postnatal growth impairs secondary ossification in the mouse tibial epiphysis
Source: Bone Res. 2019 Aug 5;7:24. doi: 10.1038/s41413-019-0064-9 (PMC6804621; doi:10.1038/s41413-019-0064-9)
Supplement: Supplementary file 2 — supplementary table 2 [file 41413_2019_64_MOESM2_ESM.docx]

**Supplementary table 2. Antibodies used for IHC**

| **Name** | **Company** | **Product #** | **Lot #** | **Source Antigen** | **Species Raised** | **Reaction Species** | **Dilution** |
| --- | --- | --- | --- | --- | --- | --- | --- |
| ALP | DSHB | B4-78 | 8/23/12-38ug/mllg | Full Length Protein | Mouse | Human, Mouse, Rat | 1:10 |
| BSP | Dr. Renny T. Franceschi | U. of Michigan | Validated antiserum | BSP | Rabbit | Mouse | 1:200 |
| Col10 | Dr. Raymond Boot-Hardford | U. of Manchester, UK | Validated antibody | Col10 | Rabbit | Mouse | 1:50 |
| MMP-13 | Novus Biologicals | NBP1-45723 | 9A213533 | Surrounding Amino Acid 454 of Rat MMP-13 | Rabbit | Human, Mouse, Rat, Bovine, Horse, Rabbit | 1:100 |
| OSX | Santa Cruz | sc22536-R | J3112 | Human SP7 | Rabbit | Mouse, Rat, Human | 1:200 |
