# Supplementary material for: Conditional disruption of the osterix gene in chondrocytes during early postnatal growth impairs secondary ossification in the mouse tibial epiphysis
Source: Bone Res. 2019 Aug 5;7:24. doi: 10.1038/s41413-019-0064-9 (PMC6804621; doi:10.1038/s41413-019-0064-9)
Supplement: Supplementary file 3 — supplementary figure 3 [file 41413_2019_64_MOESM3_ESM.docx]

**Supplementary figure 1.** **Conditional inactivation of osterix in epiphyseal chondrocytes reduces trabecular bone mass in the epiphyses in mice**. **[A]**: Representative µCT images of the tibial epiphyses of WT mice (*Osx*^flox/flox^; *Cre-ERT2*^+^ treated with corn oil) and *Osx* cKO mice (*Osx*^flox/flox^; *Cre-ERT2*^+^ treated with tamoxifen). **[B–G]**: Quantitative µCT data of the trabecular bone volume to total volume (Tb. BV/TV), trabecular number (Tb. N), trabecular thickness (Tb. Th), trabecular separation (Tb. Sp), bone mineral density, and connectivity density of the tibial epiphyses as shown in A. Values are the mean ± SEM (N = 7, 4 males and 3 females). A: a significant difference (*P* < 0.05) in *Osx* cKO epiphyses as compared to WT controls.

**Supplementary figure 2. Conditional inactivation of osterix in epiphyseal chondrocytes reduces trabecular separation in the growth plate in mice**. **[A]**: Representative µCT images of the tibial growth plate of WT mice (*Osx*^flox/flox^; *Cre-ERT2*^-^ treated with tamoxifen) and *Osx* cKO mice (Osx^flox/flox^; *Cre-ERT2*^+^ treated with tamoxifen) (N = 7, 4 males and 3 females). **[B–G]**: Quantitative µCT data of the trabecular bone volume to total volume (Tb. BV/TV), trabecular number (Tb. N), trabecular thickness (Tb. Th), trabecular separation (Tb. Sp), bone mineral density, and connectivity density of the tibial epiphyses as shown in A. Values are the mean ± SEM (N = 5, 3 males and 2 females). A: a significant difference (*P* < 0.05) in *Osx* cKO epiphyses as compared to WT controls.
