# Supplementary figures and images for: Conditional disruption of the osterix gene in chondrocytes during early postnatal growth impairs secondary ossification in the mouse tibial epiphysis
Source: Bone Res. 2019 Aug 5;7:24. doi: 10.1038/s41413-019-0064-9 (PMC6804621; doi:10.1038/s41413-019-0064-9)

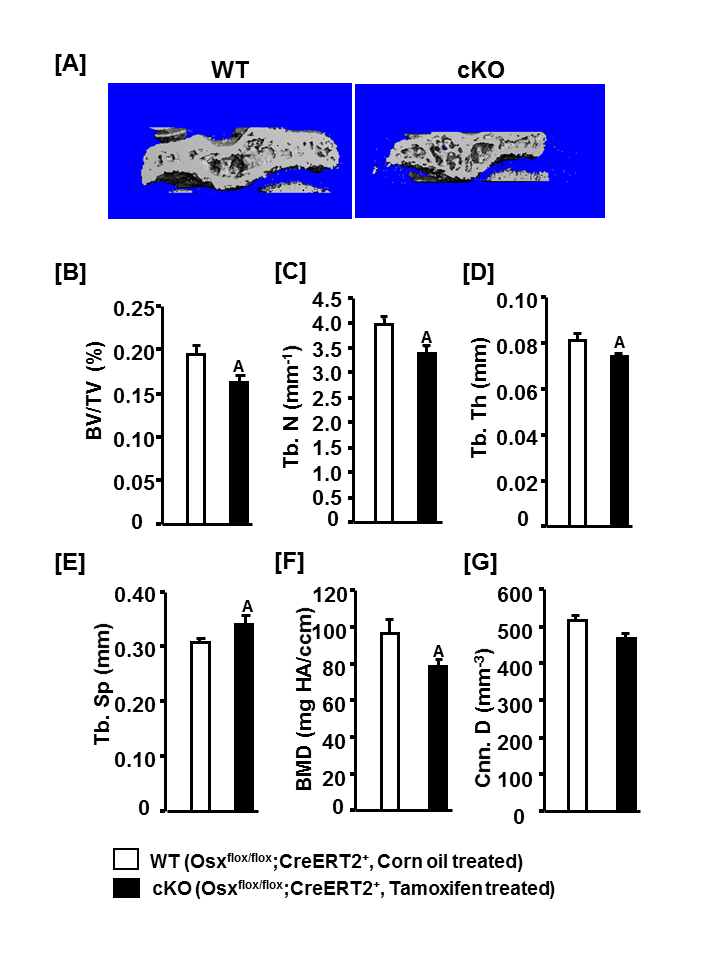

Supplement: Supplementary file 4 — supplementary figure 4 [file 41413_2019_64_MOESM4_ESM.tif]

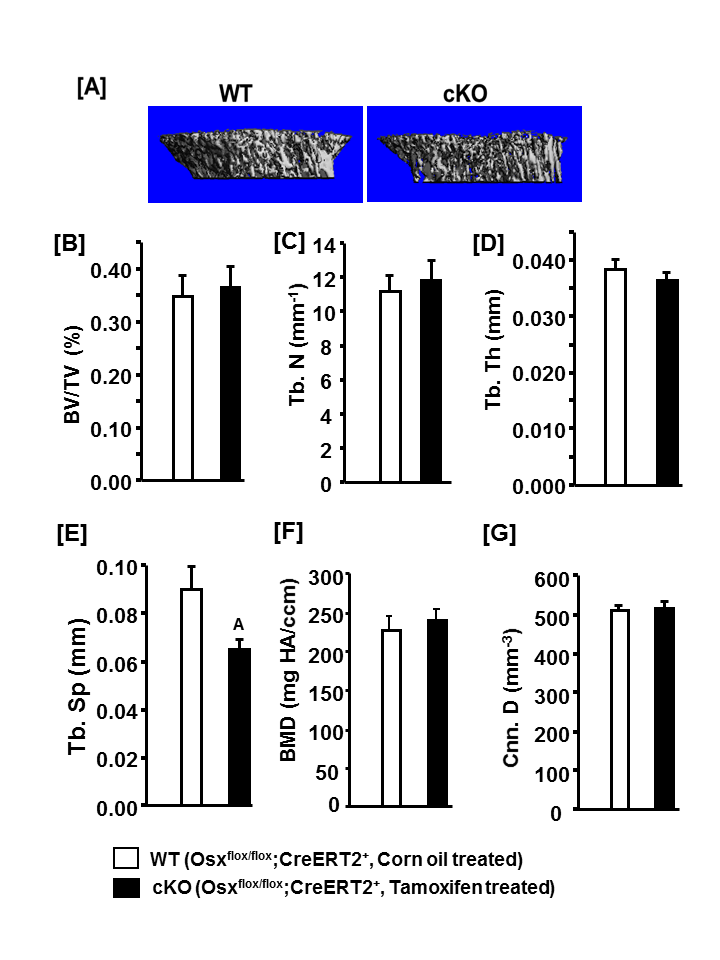

Supplement: Supplementary file 5 — supplementary figure 5 [file 41413_2019_64_MOESM5_ESM.tif]
